# Learning populations with hubs govern the initiation and propagation of spontaneous bursts in neuronal networks after learning

**DOI:** 10.3389/fnins.2022.854199

**Published:** 2022-08-18

**Authors:** Xiaoli Jia, Wenwei Shao, Nan Hu, Jianxin Shi, Xiu Fan, Chong Chen, Youwei Wang, Liqun Chen, Huanhuan Qiao, Xiaohong Li

**Affiliations:** ^1^Academy of Medical Engineering and Translational Medicine, Tianjin University, Tianjin, China; ^2^Tianjin Key Laboratory of Brain Science and Neural Engineering, Tianjin, China

**Keywords:** cultured neuronal networks, spontaneous synchronized bursts, network architecture, learning, multi-electrode array (MEA)

## Abstract

Spontaneous bursts in neuronal networks with propagation involving a large number of synchronously firing neurons are considered to be a crucial feature of these networks both *in vivo* and *in vitro*. Recently, learning has been shown to improve the association and synchronization of spontaneous events in neuronal networks by promoting the firing of spontaneous bursts. However, little is known about the relationship between the learning phase and spontaneous bursts. By combining high-resolution measurement with a 4,096-channel complementary metal-oxide-semiconductor (CMOS) microelectrode array (MEA) and graph theory, we studied how the learning phase influenced the initiation of spontaneous bursts in cultured networks of rat cortical neurons *in vitro*. We found that a small number of selected populations carried most of the stimulus information and contributed to learning. Moreover, several new burst propagation patterns appeared in spontaneous firing after learning. Importantly, these “learning populations” had more hubs in the functional network that governed the initiation of spontaneous burst activity. These results suggest that changes in the functional structure of learning populations may be the key mechanism underlying increased bursts after learning. Our findings could increase understanding of the important role that synaptic plasticity plays in the regulation of spontaneous activity.

## Introduction

Spontaneous bursts have been observed in various mammalian central nervous system neuronal types, as well as in organotypic slice cultures or various cultures of neuronal networks *in vitro* (Harris et al., 2001; Yanagawa and Mogi, 2009; Sato et al., 2012), the latter of which are thought to play a crucial role in communication in neuronal networks (Lisman, 1997), by serving as rhythm generators or creating a specific frequency component of brain waves (Wang, 2010). An increasing number of studies have indicated that bursts may play a role in network development and maturation (Baltz et al., 2010; Kilb et al., 2011), learning and memory (Pike et al., 1999; Harris et al., 2001), and information communication and processing (Ringach, 2009). However, it is unclear how spontaneous bursts develop in a network.

Several studies have investigated how spontaneous bursts are initiated in neuronal networks. Some studies have indicated that a dendritic calcium spike could be triggered when backpropagated action potentials in cortical pyramidal neurons combine with weak synaptic inputs in the apical dendrites. When the soma receives the calcium pulse, it then fires a corresponding burst (Fregnac, 1999; Grattarola et al., 2001). Several studies have suggested that the balance of excitation and inhibition is one of the most important factors inducing burst activity in a network (Kudela et al., 2003; Geng et al., 2015). The influence of other factors, such as heterogeneous delays, noises, autapses, and connection topology, in the synchronization of bursting has been demonstrated. For instance, Wang et al. (2011a,b) and Guo et al. (2012) described the mechanisms underlying time delays in synchronous and asynchronous bursts in neuronal networks. The effect of white noise on the dynamics of a delayed electrically coupled pair of Hidmarsh-Rose bursting neurons was also investigated (Buric et al., 2007). Zheng and Lu (2008) have proposed that chaotic burst synchronization is observed if the link probability and coupling strength of a small-world neuronal network are large enough. The coupling strength, as well as the probabilities of intracluster and intercluster connections, determine bursting synchronization in small-world networks (Batista et al., 2012). Computational network models show that modular network topologies with highly connected subnetworks are optimal for creating and maintaining network activity (Kaiser and Hilgetag, 2010; Klinshov et al., 2014). Several studies have indicated that the modularity of networks affects global burst synchronization (Yang and Wang, 2016; Moriya et al., 2017). However, few studies have focused on the intrinsic association between the learning phase and spontaneous bursts in networks.

To investigate the learning ability of cultured neuronal networks, researchers have constructed learning models to study network plasticity (Shahaf and Marom, 2001; Pimashkin et al., 2013). Shahaf and Marom (2001) found that repeated cycles of learning ultimately led to a significantly improved stimulus response. Li et al. (2007) studied the effect of learning on spontaneous bursts by constructing a learning model. They found that learning enhanced the firing, association and synchrony of spontaneous burst activities in the neuronal network. Bologna et al. (2010) found that low-frequency stimulation enhanced burst activity in cortical cultures. Le Feber et al. (2010) found that a learning protocol induced functional connectivity changes. Moreover, they hypothesized that networks developed a balance between connectivity and activity (Le Feber et al., 2010). The combination of applied stimulus and initial connectivity induced changes in connectivity (Le Feber et al., 2010). However, there are few reports on the mechanism underlying learning-encoding bursts in cultured neural networks.

Electrophysiological signals are recorded by microelectrode arrays (MEAs). MEAs with 60–250 electrodes represent a well-established technology, as demonstrated for recording network-wide extracellular activity from neuronal cultures prepared from rodents; for studying the developing patterns of network activity and to investigate the network responsiveness to electrical stimuli. However, the spatial resolution of a traditional MEA is unable to observe the propagation of spontaneous activity patterns over large neuronal networks with high spatial resolution, which could lead to the misestimation of population parameters (Gerhard et al., 2011; Ribeiro et al., 2014). Furthermore, if networks are large enough, they can enter a state of continuously circulating synchronous bursting activity (Keren and Marom, 2016). The burst propagation pattern of neuronal networks recorded by low-density MEAs showed that most of the onset regions of synchronous bursts originated outside the recording array (Okujeni et al., 2017; Pasquale et al., 2017). Therefore, a high-density MEA (HD-MEA) is required to record the propagation patterns of spontaneous bursts. A HD-MEA with 4,096 recording electrodes provides exceptional spatiotemporal resolution for exploring spontaneous activity of neuronal networks *in vitro*.

In this study, to investigate the mechanism underlying changes in burst activity due to learning, we located a “learning population” that encoded stimulus information in the network. To assess network plasticity and learning stability, we evaluated whether plasticity and stability reflects learning in the learning population. Then, we investigated the relationship between burst initiation after the learning phase and the learning population. We found that learning population, which encodes stimulus information, gradually formed a functional network with the ability to process information during the learning phase and that the learning population with the largest betweenness centrality in the functional network governed the initiation of spontaneous burst activity. Finally, we validated our results by manipulating the excitatory-inhibitory balance of the network with bicuculline.

## Results

### High-density recording of spontaneous activity in cultured neuronal networks

**FIGURE 1:**
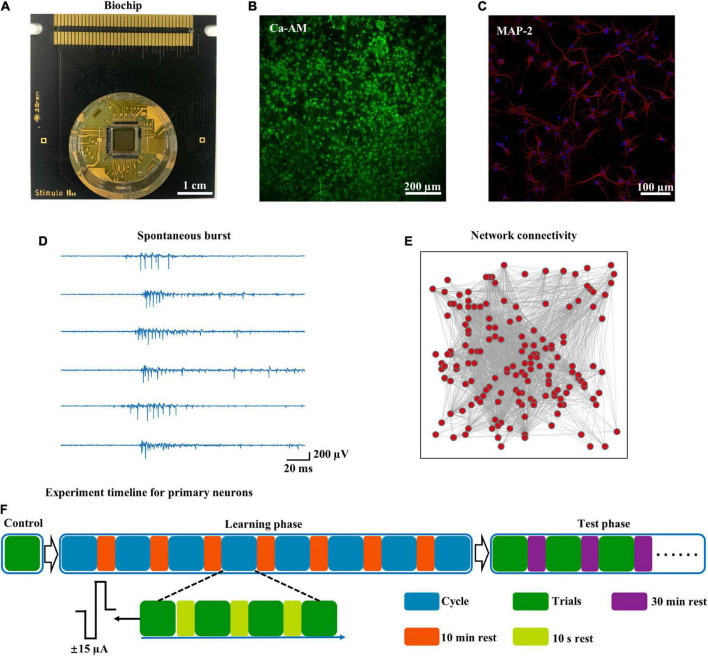
High-density recording of spontaneous activity and experimental workflow. **(A)** Overview of the HD-MEA biochip, including 4,096 recording electrodes and 16 stimulus electrodes. **(B)** The neurons on the biochip were stained with Ca-AM and photographed under confocal microscopy [at 7 days *in vitro* (DIV)]. **(C)** The same batch of neurons cultured in a culture dish was stained with MAP-2 (DIV 14): neurons (red), nucleus (blue). **(D)** Raster of spontaneous spikes in cultured neuronal networks (time: 60 s). **(E)** Representative functional network diagram of spontaneous activity (time: 60 s). **(F)** Experimental workflow for the learning phase and test phase.

An HD-MEA with 4,096 electrodes was used to record spontaneous activity from five cortical networks (Figure 1A). The neuronal network was formed (Figure 1B), and MAP-2, an axo-dendritic marker, was used to identify neurons (Figure 1C). This neuronal network generated spontaneous synchronized bursts, characterized by an intense series of spikes over a short period (Figure 1D). From an early stage [10 days *in vitro* (DIV)], synchronized bursts of cortical neurons were observed; these bursts remained stable throughout development (Kamioka et al., 1996). We computed a cross-correlation matrix to construct a functional network (nodes: electrodes, edges: correlations), which demonstrated complex spatiotemporal patterns, in the cultured neuronal network (Figure 1E). These results were consistent with a previous study that found that cultured neuronal networks were maturity and functional (Colombi et al., 2016; Dias et al., 2021). The experimental protocol (see the Materials and Methods for more details) consisted of the learning and test phases (Figure 1F). Biphasic current stimuli (200 μs in duration, amplitude ± 15 μA) were delivered at a frequency of 1 Hz from 2 spatially distant sites that had the greatest correlation in spontaneous activity.

### Learning in networks of cortical neurons

To locate the learning population in the cultured neuronal network, we initially used previously reported methods and indicators to induce learning (see the Materials and Methods for more details) (Shahaf and Marom, 2001; Li et al., 2007; Le Feber et al., 2010; Hamilton et al., 2015). For cultured neuronal networks, the stimulus can be used as a learning paradigm, causing changes in the neuronal network according to synaptic plasticity. In other words, the neuronal network adapts to the electrical stimulus. The neuronal network exhibits increasing mastery of the external input during learning. The response of the neuronal networks to stimuli can be gradually enhanced and stabilized (Shahaf and Marom, 2001). The response/stimulus (R/S) ratio is used to quantify this phenomenon. The R/S ratio refers to the proportion of 10 stimuli that elicit the desired response; thus, its value ranges between 0 and 1. In addition, the neuronal network gradually mastered the information carried by the stimulus and established the optimal path to the external stimulus during learning. The fastest path of specific external information transmission was selected and stabilized after exploration (Marom and Shahaf, 2002). This phenomenon can be quantified by the response time (RT) of neurons to external stimuli (Li et al., 2007). The R/S ratio and RT were used to evaluate learning in the cultured neuronal network.

**FIGURE 2:**
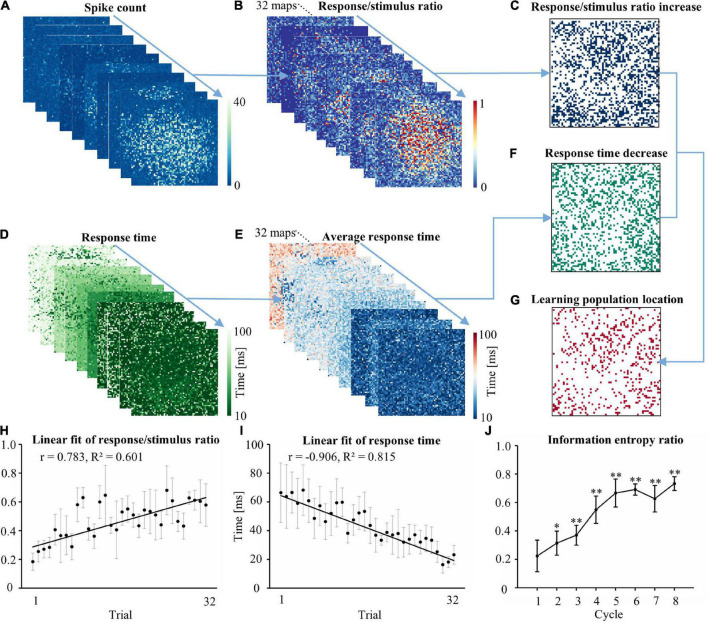
Locating learning populations on the MEA. **(A)** The response map of each electrode in the network to the stimulus in a typical trial, which consisted of 10 stimuli. **(B)** The 10 representative response maps from each trial were used to produce an R/S ratio map. The experiment consisted of 32 R/S ratio maps. **(C)** First-order linear modeling was performed on each electrode in the 32 R/S ratio maps to identify the spatial position where the slope of the fitted line was greater than 0. **(D)** The RT map of each electrode in the network to the stimulus in a typical trial. **(E)** The 10 representative RT maps from each trial were used to produce an average RT map. **(F)** First-order linear modeling was performed on each electrode in the 32 average RT maps. The spatial position where the slope of the fitted line was less than 0 was identified. **(G)** The spatial location of overlap between C and F was determined as the spatial position of the learning population in the cultured neuron network. The location of the overlap reflected the exact electrode position. **(H)** Linear regression of trial vs. the R/S ratio of the learning populations. **(I)** Linear regression of trial vs. RT for the learning populations. **(J)** The ratio of the information entropy of the learning population accounted for the total information entropy in the cultured neuronal network in each cycle (*n* = 5 cultures. Data collected during 10–200 ms after the stimulus were analyzed. *p < 0.05 vs. Cycle 1, **p < 0.01 vs. Cycle 1. R/S: response/stimulus, RT: response time).

We calculated the number of spikes during 10–200 ms following each stimulus. Increased evoked firing was observed following the stimulus after learning. Figure 2A shows the number of spikes following the stimulus in a trial. The evoked response of each electrode in a trial to the 10 stimuli was used to generate a graph of the R/S ratio. First-order linear modeling was performed on the R/S ratio of 32 trials during one experiment (Figure 2B). The electrode location with a positive slope in the linear fitting was defined as the electrode with a continually increasing R/S ratio (Figure 2C). Figure 2D shows the representative RT after stimulus in a trial (each trial contained 10 stimuli). The RT of each electrode in a trial was averaged to obtain the average RT (Figure 2E). The average RT of the 32 trials in the experiment was fitted by first-order linear modeling to acquire the electrode position that exhibited decreasing RT (Figure 2F). The spatial overlap in Figures 2C,F represented the location of the learning populations (Figure 2G). Figures 2H,I show the fitting result of the R/S ratio and RT of the learning populations in the experiment. The R/S ratio of the learning populations (Figure 2H) gradually increased (*r* = 0.783, *R*^2^ = 0.601), and the RT (Figure 2I) gradually decreased (*r* = −0.906, *R*^2^ = 0.815) during the experiment. Thus, successful learning was induced in the networks. To assess the ability of the learning populations to carry information during the learning phase, we calculated the information entropy of these populations (Figure 2J). We found that the information carried by the learning populations gradually increased, and learning populations were able to recover approximately 75% of the information gained in the global population (defined as the electrophysiological signals of all recording electrodes).

**FIGURE 3:**
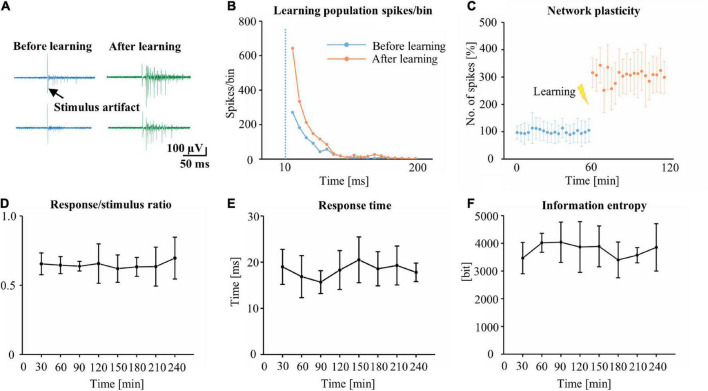
The identification of learning and network plasticity. **(A)** The original trace represents the typical stimulus-evoked responses before and after training. **(B)** A comparison of typical stimulus-evoked responses of learning populations within 200 ms of the stimulus, before and after learning (*n* = 349 learning populations, bin = 10 ms). **(C)** Time course of the number of spikes in the stimulus-evoked responses of learning populations before and after learning (time: 120 min, 100% represents the average before training for 60 min). The R/S ratio **(D)**, RT **(E)** and information entropy **(F)** of learning populations during the test phase (*n* = 5 cultures. R/S: response/stimulus, RT: response time).

To understand the changes in the plasticity of cultured neuronal networks, we applied a stimulus to a pair of channels and analyzed the stimulus-evoked response to the test stimulus every 1 min before and after 60 min. Examples of stimulus-evoked responses in the cultured neuronal network are shown in Figure 3A. Following learning, the response was increased in the 200 ms after the stimulus. Figure 3B shows typical stimulus-evoked responses of an increased number of spikes in learning populations. Figure 3C shows the increasing firing rate after learning. The learning population’s average firing rate for 1 h after learning was increased by 217% compared with that before learning. To test the learning stability of learning populations in the network, the same electrical stimulus as that applied during the learning phase was applied to the network every 30 min following learning during the test phase (total duration: 240 min), and the stimulus-evoked response of the learning populations was analyzed. During the test phase, the R/S ratio (Figure 3D), the RT (Figure 3E), and the (Figure 3F) of the information entropy learning populations were maintained in a stable range, suggesting that learning populations indeed contributed to recognizing the stimulus.

### The initiation and propagation of spontaneous burst activity changed after learning

**FIGURE 4:**
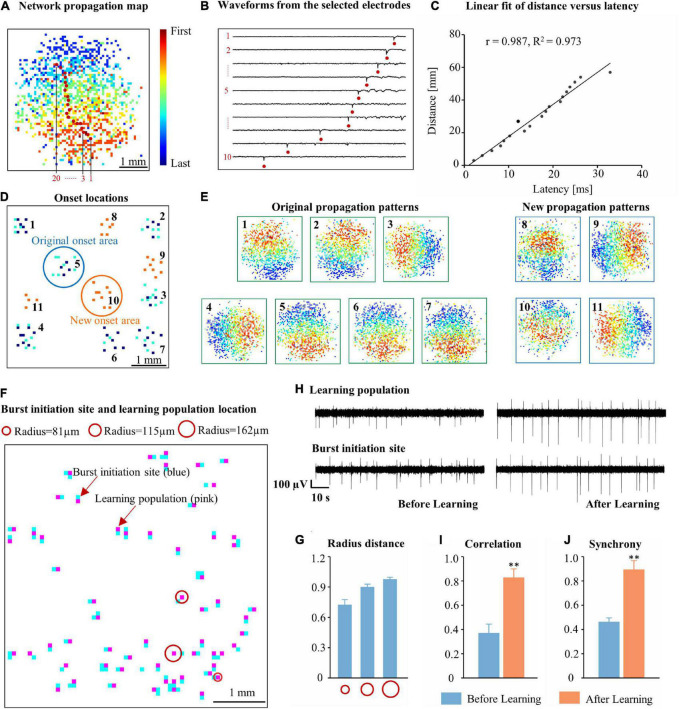
Initiation and propagation of spontaneous burst activity changed after learning. **(A)** A typical network burst propagation map. The propagation patterns were revealed by mapping the rank order of the first spike on each electrode during spontaneous synchronized bursts. The propagation pattern from the initial regions of the burst to outside of the area is marked with red dots. Color codes indicate the rank order of the first spike on each electrode during spontaneous bursts. **(B)** Waveforms from the first selected 10 electrodes in **(A)**. **(C)** Linear regression of distance vs. latency for the selected electrodes in **(A)**. **(D)** Representative diagram of the distribution of burst onset in a culture within 5 min before and after learning (blue dots: before learning, orange and cyan dots: after learning). The representative original burst onset area is circled in blue, and the new onset area is circled in orange. **(E)** Representative burst propagation pattern in each burst area (the number code follows that in **D**). **(F)** The distance between the burst initiation site and location of learning populations (for 67 burst initiation sites). **(G)** The distances between the burst initiation sites and the learning populations from **(F)**. **(H)** Raw voltage recording of burst initiation sites and learning populations before and after learning. The correlation **(I)** and synchrony **(J)** between burst initiation sites and learning populations (time = 60 s, *n* = 5 cultures. *p < 0.05 vs. Cycle 1, ^**^p < 0.01 vs. Cycle 1).

We found that the number of spontaneous synchronized burst of the network within 1 min increased significantly after the learning phase (Supplementary Figure 1A, *P* < 0.01); the number of electrodes participating in each burst showed increased significantly (Supplementary Figure 1B), and the duration of the spontaneous synchronized bursts also increased slightly (Supplementary Figure 1C). Were these increased bursts merely repetitions of the existing burst pattern or did learning induce a new pattern? To address this question, we used first spike rank-order maps to visualize initiation and propagation of spontaneous bursts (Figure 4A). We defined the first ten recruitment ranks as the onset electrodes of a spontaneous burst. The initiation site of the burst was determined, and the path of the raw waveform (from inside to outside) was drawn (Figure 4B). The selected electrode spike timestamp and distance from the initiation site were fitted to a model (Figure 4C, *r* = 0.978, *R*^2^ = 0.973). The results of the network propagation map showed that the burst originated from a localized network region and recruited other parts of the network. New burst initiation areas and burst propagation patterns appeared in the network after learning (Figures 4D,E). Therefore, electrical stimuli induce changes in network burst initiation regions and propagation patterns.

Next, to explore new burst initiation regions in the network after the learning phase, we calculated the distance between the average positions of the first 10 burst initiation sites in the network burst propagation map as well as the nearest learning population (Figure 4F, recording time: 10 min). The distance between the centers of the two electrodes was 81 μm. We were surprised to find that 72.42% of the average initiation electrode positions were 81 μm away from learning populations, 90.02% of the average initiation electrode positions were within 115 μm of learning populations, and 97.64% of the electrode positions were within 162 μm of learning populations (Figure 4G). These findings demonstrated that the burst initiation region was intrinsically linked to learning populations. To assess this, we examined the firing pattern before and after the learning phase. Before learning, the firing pattern between the burst initiation site and the learning population was quite different; in contrast, after learning, the electrophysiological signals at these same electrodes were highly synchronized (Figure 4H). The correlation and synchrony between the learning population and burst initiation sites after learning was significantly higher than before learning (Figures 4I,J). Therefore, we speculate that the emergence of new bursts in the cultured neuronal network after learning was likely predominantly due to learning populations.

### Learning populations with hubs govern the initiation of spontaneous bursts after learning

**FIGURE 5:**
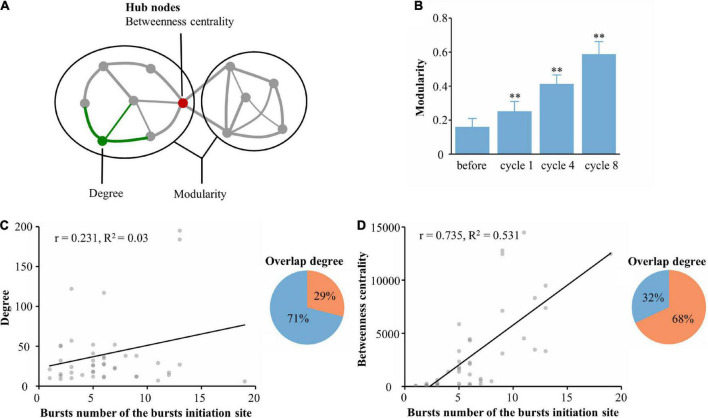
Generation of spontaneous network bursts in the functional learning populations. **(A)** An artificial network example, where degree represents the number of links connected to a node (green) or module (ovals). Hubs (red) often occur along the shortest paths and consequently often have a high betweenness centrality. **(B)** The network modularity was compared throughout the experiment during spontaneous activities. **(C)** Linear regression of the number of bursts at burst onset and the average degree of the learning populations within 115 μm of the burst onset (left). The overlap between neurons involved in the burst onset and those in the learning population with the top 90% modularity is shown in the right pie chart as orange. **(D)** Linear regression of the number of bursts at burst onset and the average betweenness centrality of learning populations within 115 μm of the burst onset (left). The overlap between neurons involved in the burst onset and those in the learning population with the top 90% betweenness centrality is shown in the right pie chart in orange (*n* = 5 cultures, time: 5 min, *n* = 284 bursts. *p < 0.05 vs. before, ^**^p < 0.01 vs. before).

Synchronous burst activity has been reported to depend on network modularity (Yang and Wang, 2016; Moriya et al., 2017). Therefore, we investigated the possible mechanisms underlying spontaneous burst generation associated with functional communities after learning. We hypothesized that the functional network consisting of learning populations cooperatively processes information during the learning phase, which changed the modularity of the network and ultimately led to a change in the burst pattern of spontaneous activity after learning. In networks, a module is a subset of highly connected nodes; modularity is a metric that measures the degree of modularization (Rubinov and Sporns, 2010; Figure 5A). The degree of modularization of the functional network constructed by the learning populations was calculated. As shown in Figure 5B, the degree of modularization increased significantly with learning. Next, we wanted to know which characteristic in the learning population led to the emergence of new burst patterns. We constructed a linear model of the number of bursts at burst onset and the learning population within 115 μm of the burst onset with an average degree of modularization (*r* = 0.231, *R*^2^ = 0.03, 5 cultures, time: 5 min, *n* = 284 bursts, Figure 5C). We then calculated the degree of overlap between the burst onset and neurons with the top 90% modularity in the learning population; there was only 29% overlap between neurons involved with burst onset and the learning population. To further investigate whether the hubs (quantified by betweenness centrality) could be a key feature of the functional network, we repeated the same quantification between the number of bursts at burst onset and the learning population. We found a clear positive correlation between the burst number and betweenness centrality of neurons (*r* = 0.735, *R*^2^ = 0.531, 5 cultures, time: 5 min, n = 284 bursts, Figure 5D). We also calculated the overlap degree between the burst onset and the neurons with the top 90% betweenness centrality in the learning population; there was up to 68% overlap between neurons involved with burst onset and the learning population. From these results, we fully confirmed our hypothesis; moreover, we propose that learning populations with higher betweenness centrality in the functional network govern the initiation of spontaneous burst activity after learning.

**FIGURE 6:**
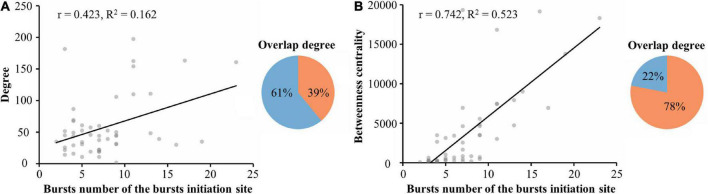
Altering the excitatory-inhibitory balance allowed further exploration of the relationship between spontaneous bursts and hubs. **(A)** Linear regression of the number of bursts at burst onset and the average degree of the learning population within 115 μm of the burst onset (left). The overlap between neurons involved in the burst onset and those in the learning population with the top 90% modularity (right). **(B)** Linear regression of the number of bursts at burst onset and the average betweenness centrality of the learning populations within 115 μm of the burst onset (left). The overlap between neurons involved in the burst onset and those in the learning population with the top 90% betweenness centrality (right) (*n* = 5 cultures, time: 5 min, *n* = 387 bursts).

Reports have indicated that changes in the balance between excitatory and inhibitory synaptic activity contribute to increased network burst activity (Kudela et al., 2003). To confirm this hypothesis, we manipulated the network excitation-inhibition balance with bicuculline, a gamma-aminobutyric acid A (GABA A) receptor-specific blocker that exhibits dose-dependent effects (Teppola et al., 2019). We found that learning populations were more sensitive than global populations to changes in the network state (Supplementary Figures 2, 3). We also found that the number of spontaneous bursts per minute increased after adding bicuculline (Supplementary Figure 3H), and the number of electrodes participating in each burst also increased significantly (Supplementary Figure 3I). Additionally, burst duration was significantly prolonged (Supplementary Figure 3J). After adding bicuculline, the correlation between the number of bursts at burst onset and the average betweenness centrality of the learning population was significant and positive (*r* = 0.423, *R*^2^ = 0.162, 5 cultures, time: 5 min, *n* = 387 bursts) as well as higher than the average degree fitted (*r* = 0.742, *R*^2^ = 0.523, 5 cultures, time: 5 min, *n* = 387 bursts), as shown in Figure 6. There was up to 78% overlap between the neurons involved with burst onset and the betweenness centrality of the learning population. This result was robust across the different cultured neuronal networks, suggesting that learning populations with higher betweenness centrality in a functional network govern the initiation of spontaneous burst activity after learning; and that this pattern is a fundamental property of the network.

## Discussion

Using an HD-MEA, we recorded the firing activity of large-scale networks. Our results indicated that learning populations represented the learning ability of cultured neuronal networks and carried most of the stimulus information. We reported that the learning population gradually formed a functional network with the ability to process information during the learning phase, and the learning population in the functional network with the largest betweenness centrality governed the initiation of spontaneous burst activity. Our results suggest that network hubs could play an important role in spontaneous bursts after learning. Functional connectivity as well as spontaneous burst generation and propagation may be particularly important to understand mechanisms of neural information processing.

The use of HD-MEAs is an advanced technique for recording the electrophysiological activity of neuronal networks with a cellular-level spatial resolution and a single spike-level temporal resolution (Imfeld et al., 2008; Berdondini et al., 2009). Studies have found that only a small fraction of neurons exhibit high excitability (Wohrer et al., 2013), and these neurons are likely to play critical functions in the network (Panas et al., 2015); overlooking these neurons could lead to misinterpretation of our results. To prevent such sampling bias, the measurements must possess a cellular-level spatial resolution. Moreover, because these spontaneous bursts last only a few hundred milliseconds, the temporal resolution must be at the millisecond level to accurately identify the propagation pattern of bursts (Eytan and Marom, 2006). To the best of our knowledge, HD-MEAs are the ideal device for simultaneously achieving these temporal and spatial resolutions. In our experiment, we implanted ∼90,000 neurons in a 5.12 × 5.12 mm^2^ electrode area to form a network. These 4,096 electrodes and ∼90,000 neurons produced a sampling ratio of approximately 1:22, allowing us to evaluate sustained slight changes in all regions of the network. Given the 1:22 sampling ratio, we may have overlooked the electrophysiological signals of crucial yet infrequent neurons. However, the single neuron resolution provided by the HD-MEA was sufficient to record the initiation and propagation patterns of neuronal populations during bursts (Lonardoni et al., 2017). Yada et al. (2016a,b) studied the repeating spatiotemporal patterns of network spontaneous bursts with HD-MEAs. Okujeni et al. (2017) and Okujeni and Egert (2019a,b) researched the initiation and propagation of spontaneous bursts using MEAs with 1,024 electrodes. Therefore, HD-MEAs are an ideal experimental tool for studying the mechanism of spontaneous network bursts.

We applied low-frequency stimulation to the cortical culture network by analyzing the R/S ratio and RT in open-loop conditions. For cultured neuronal networks, an electrical stimulus can be used as a learning paradigm, causing changes in a neuronal network according to its plasticity (Shahaf and Marom, 2001). Notably, not all cultures demonstrate plasticity; therefore, we chose cultures that matched the following criteria (Chiappalone et al., 2008): (i) cultures that were spontaneously active in terms of spiking and bursting activity, (ii) a network that responded to electrical stimulation, and (iii) a stable mean firing rate. Previous studies have used the R/S ratio and RT to evaluate the learning ability of cultured neuronal networks (Li et al., 2007; Chiappalone et al., 2008). We used these values to identify the spatial location of learning populations in the cultured neuronal network. During the learning phase, the R/S ratio of the learning populations gradually increased, and the RT gradually decreased, indicating that learning occurred in the cultured neuronal network. Previous research has shown that this type of learning is extremely selective. Only the neurons on the trained electrodes increase the quantity of spikes in response to the stimulus, resulting in a higher R/S ratio (Stegenga et al., 2009). In our experiments, we also found an increase in the response from learning populations. Another important characteristic is how long synaptic modifications in the network are sustained after learning. Our study revealed that learning populations exhibited network plasticity and stable responses to the stimulus within 6 h of learning. The network showed long-term plasticity, consistent with previous results (Pimashkin et al., 2013). Thus, stimulation in open-loop conditions may induce long-term changes in the structure and function of cultural networks, including synaptic connectivity. Previous studies have used the first 10 spatial principal components to locate the small subset of neurons that carries most of the stimulus information in the network (Angotzi et al., 2019); the most informative neurons carried nearly enough information to support the discrimination abilities of the entire animal. In our study, the information carried by learning populations during the learning phase gradually increased and eventually accounted for approximately 80% of the information in the global network. This result was consistent with Nieus’s findings that a small population of neurons carries most of the stimulus information in the network (Nieus et al., 2018).

We found that learning enhanced the firing of spontaneous bursts in neuronal networks, consistent with a previous study showing that successful learning can drive neuronal activity and improve the connection and synchrony of spontaneous firing in neuronal networks (Li et al., 2007). We found that the strength of a large portion of functional connections changed, as exhibited by comparing spontaneous activity before and after learning. Therefore, the learning phase influenced functional network connectivity. One explanation for successful learning is the balance between activity and connectivity that cultured neuronal networks develop (Van Pelt et al., 2004). The stable activity patterns of networks may be interpreted as an established balance between activity and connectivity (Le Feber et al., 2007). If external stimulation drives the network off balance, it may achieve a dynamic balance that includes or excludes a given connection (Le Feber et al., 2010). Thus, stimuli might trigger internal network forces that induce connectivity changes. Vajda et al. (2008) stimulated a cultured network with a low-frequency stimulus and thereby induced changes in stable patterns of spontaneous activity, as observed through changes in a single site and culture-wide spontaneous burst activity in the network. They proposed that changes in connectivity caused by low-frequency electrical stimulation may be caused by (1) plasticity, (2) altered intrinsic neuronal characteristics such as excitability, or (3) transition from one attractor state to another.

The mechanisms and network structures underlying spontaneous burst initiation and propagation in cultured neuronal networks remain unclear. A previous study showed that protein kinase C can change the balance between local and long-range connectivity, and clustering enhanced spontaneous burst generation (Okujeni et al., 2017). Previous works have indicated that spontaneous bursts are preceded by the activation of a subset of overactive electrodes (Eytan and Marom, 2006; Eckmann et al., 2008). Our results suggest that the activity of learning populations gradually increases during the learning phase and that this activity is retained in spontaneous activity. Other studies confirmed that bursts and phase profiles change during conditions of repeated stimulation (Stegenga et al., 2009). We also found that the burst propagation pattern changed after learning. Alternatively, pacemaker neurons or highly active neurons (known as “leader sites,” “major burst leaders” or first-to-fire neurons) may play a role in spontaneous burst initiation (Eytan and Marom, 2006; Eckmann et al., 2008; Ham et al., 2008; Shein et al., 2008; Pasquale et al., 2017). Studies have argued that early-firing “leader” sites are part of a subnetwork that is consistently excited during the initial stages of activity propagation (Yada et al., 2016b). However, we found that the firing pattern of learning populations after learning and the firing pattern of the burst initiation sites showed a high correlation and synchronization. Therefore, we speculated that the new bursts after learning were related to learning populations. Learning populations function in a similar manner as the “leader” sites of the network, namely, both are reliably and rapidly recruited in spontaneous and evoked firing patterns.

Finally, by analyzing the properties of the functional network, we found that hubs in the functional network were highly coincident with the burst onset. This result implies that functional network structure and spontaneous bursts are intrinsically related. These learning populations were dispersed throughout the network and were characterized by strong functional connections with low average path lengths. Previous studies have reported that neuronal networks exhibit a small-world topology, with a short mean path length and a strong clustering coefficient (Watts and Strogatz, 1998; Yu et al., 2008). Furthermore, hubs, or groupings of neurons with a high out/in degree, are common in these neural networks, allowing information to be transported effectively (Bettencourt et al., 2007). Recently, hubs in cultured neuronal networks have been suggested to be involved in propagating spontaneous activity from first-to-fire neurons to the global network (Schroeter et al., 2015). Previous studies have also suggested that the spontaneous bursts of networks are intrinsically related to their modularity (Moriya et al., 2017; Yang et al., 2017). Our findings were consistent with those of earlier research. Taken together, these results suggest that learning populations gradually become functional networks during the learning phase due to their highly organized spatial structure, graph-theoretic properties, and strong connectivity; these functional networks may act as hubs that govern spontaneous burst activity.

## Materials and methods

### Cell culture and data acquisition

The biochip was coated with laminin (0.1 μg μL^–1^; Sigma, United States) (3 h) and poly-D-lysine (0.1 μg μL^–1^; Sigma, United States) (overnight) and then rinsed with sterilized water. Primary cortical cells were obtained from the brain tissue of Sprague–Dawley (SD; Charles River, China) rats at embryonic day 18 (E18) following protocols reported in previous works (Rasband, 2010). Briefly, embryos were removed and dissected under sterile conditions. The cortex was dissociated by enzymatic digestion in 0.125% trypsin (Thermo Fisher Scientific, United States) for 10 min at 37°C and finally triturated with a Pasteur pipette. Dissociated neurons were plated on the active area of the biochip. To cover the entire active area (5.12 mm × 5.12 mm), we used drops of various volumes of cell suspensions (ranging from 80 to 90 μL) and variable cell concentrations (∼1,000–1,500 cells μL^–1^). Four hours later, when cells had adhered to the substrate, 1.5 mL of medium was added to the biochip. The cells were incubated with neurobasal medium (Invitrogen, United States) supplemented with 1% GlutaMAX (Gibco, United States) and 2% B-27 plus (Gibco, United States) in a humidified atmosphere of 5% CO_2_ at 37°C. Before starting an experimental session, we waited for approximately 30 min to allow the cultures to stabilize after removal from the incubator (Streit et al., 2001). All experiments were performed on cortical cultures (at 21–27 DIV), by which time neurons were expected to have matured and be electrically recognizable. We used 10 neuronal cultures obtained from pups from 8 different rats. Of these, 2 cultures were excluded because of uneven neuronal distribution, and 3 cultures were excluded because their firing rates did not meet the basic requirements. This experiment was approved by the Animal Ethical and Welfare Committee of Tianjin University.

We performed all electrophysiological signal recordings using the BioCam X system (3Brain, Switzerland). The biochip contains an array of 4,096 recording electrodes and 16 stimulation electrodes uniformly distributed on the active area (21 × 21 μm^2^ in size, 81-μm pitch) on an active area of 5.12 × 5.12 mm^2^, centered in a working area of 6 × 6 mm^2^. We used BrainWave 4 software (3Brain, Switzerland) for data recording and spike detection. Data were analyzed through MATLAB. Data are expressed as the means ± standard errors of the mean (SEs). The *t*-test was used to detect significant differences between two groups. *P* < 0.05 was considered statistically significant.

### Experimental protocol

We used balanced negative-first biphasic current pulses for the electrical stimulation because of their effectiveness (Odawara et al., 2016). The current pulses had phase durations of 200 μs and amplitudes between ± 15 μA. A stimulus was applied to one pair of electrodes at a time in all stimulus protocols; the two stimulus electrodes (out of the 16 stimulus electrodes) were selected as the pair with the greatest correlation in spontaneous firing activity, i.e., the stimulus electrodes at (3, 3) and (2, 2). The stimulus was applied at a frequency of 1 Hz, which was chosen because of its possible effect on intrinsic neuronal plasticity and learning (Li et al., 2007). A total of 4,096 recording electrodes simultaneously recorded the electrophysiology signals. The basic experimental protocol consisted of control, learning and test phases (Shahaf and Marom, 2001; Li et al., 2007; Pizzi et al., 2009; Pimashkin et al., 2013). We applied the stimulus to a pair of channels and analyzed the response to the test stimulus every 1 min for 60 min during the learning phase. The purpose of the learning phase was to allow neurons to gradually adapt to the electrical stimulus and respond stably to the electrical stimulus at a fixed position. The learning phase was followed by a test phase in which the culture was stimulated with a similar stimulus and its response for further analysis. The learning phase was divided into 8 cycles, each containing 4 trials; each of the trials contained 10 stimulations (10 s). After each stimulus trial, the neurons rested for 20 s; after each cycle, the neurons were allowed to rest for 1 min. A total of 320 stimuli were administered to the neural network during the learning phase. The neural networks were tested after the learning phase with a 20-min test phase. During the test phase, the same position in the neural network was stimulated 10 times, once every 0.5 h. Bicuculline, a specific antagonist of the GABA A receptor, was applied to the network to verify the characteristics of the dynamic change. First, the networks underwent the learning phase, and learning populations were successfully detected. Then, the cultured neuronal networks were tested, and their response signals were recorded after the addition of 50 μM bicuculline. The cultured neuronal networks were tested again after the bicuculline was washed out. The whole experiment lasted approximately 8 h.

### Immunohistochemistry and fluorescence imaging

After the experiments, neurons were fixed in 4% paraformaldehyde (Invitrogen, United States) in PBS (phosphate-buffered saline; Sigma, United States) and permeabilized (0.25% Triton X-100, Sigma, United States) in PBS. Then, the primary antibody against MAP2 (Abcam, United Kingdom) was diluted 1:500 in PBS with 1% BSA (bovine serum albumin; Sigma, United States); 0.1% Tween 20 was added the solution was left overnight at 4°C on a shaker at low speed. The secondary antibody conjugated with Alexa Fluor 647 (Invitrogen, United States), diluted to 1:200, was applied for 1 h at room temperature in the dark (Bakkum et al., 2013). Laser scanning confocal microscopy (Nikon, Japan) was performed on the neuronal network after loading with 2 μM calcein (Sigma, United States) green AM for 15 min at room temperature in HBSS (Thompson et al., 2006). Neurons were monitored for at least 10 min after the experiments to ensure that the calcein fluorescence was stable.

### Spike detection and burst detection

Spike detection analysis was performed by employing the Precise Timing Spike Detection (PTSD) algorithm (Maccione et al., 2009) integrated in the Brainwave software application (3Brain, Switzerland). A threshold of 8 times the standard deviation was used for spike detection. All spike trains were exported from BrainWave to MATLAB files and were analyzed with custom MATLAB scripts. We calculated the spike triggered-average (STA) for a single electrode by dividing the median by many time-aligned spike occurrences (Muller et al., 2015). Burst detection was based on previously reported methods (Vajda et al., 2008). Network bursts were defined as more than 25% (>1,024) of active electrodes in the network firing spikes synchronously in the same bin.

### Identification of the spatial location of learning populations

The location of learning populations was identified with low-frequency stimulation. A stimulus-evoked response was defined as instances when the number of spikes on the electrode exceeded the average number of spikes in the cultured neuron network. The detection of the stimulation artifacts was afforded with a hard threshold (Olshausen, 2003). The first 10 ms after the stimulus were discarded in order to avoid any possible effect of the stimulus artifact. The RT was defined as the average of the timestamps of the first 3 spikes after stimulation (analysis interval: 10–200 ms after stimulus). The R/S ratio refers to the proportion of 10 stimuli that reach the desired response (the R/S ratio gradually increases during the learning phase); thus its values range from 0 to 1. If the RT decreased gradually in eight cycles, the networks exhibited learning. Similarly, if the R/S ratio increased gradually in eight cycles, the network was also considered to exhibit learning (Shahaf and Marom, 2001). The RT and R/S ratio were calculated for each electrode, and first-order linear modeling was performed to determine the spatial position of the neuron where the R/S ratio gradually increased and the RT gradually decreased. Based on the R/S ratio and RT, we defined these electrodes as the learning population.

### Information entropy

To quantify the information carried by learning populations, we used information entropy measures. First, we computed the inter-spike interval (ISI) based on the spike train. Second, we calculated the probability of ISI in each bin. This probability value was used in the following formula to obtain information entropy.


(1) $$E=-\sum_{i=1}^{\text{n}}P_{i}\;\log_{2}P_{i}\;\left(i=1,2,\ldots ,n\right)$$

### Functional connectivity analysis of spiking activity

We used a cross-correlation algorithm based on the spike trains of learning neurons to estimate functional connectivity in networks. The method describes the network topology as a graph where nodes represent the learning neurons and edges represent structural connections. The edge weights were provided by the degree of correlation. In principle, stronger correlations between two nodes were reflected by higher weights (Ullo et al., 2014). The cross-correlation (bin = 1 ms) of spike trains was calculated between every two neurons. The following cross-correlation function was used to assess the spike trains of each pair of electrodes (x, y) (Ullo et al., 2014):


(2) $$C_{xy}\left(\tau \right)=\frac{1}{\sqrt{N_{x}N_{y}}}\sum_{s=1}^{N_{x}}\sum_{t_{i}=\tau -\left(\Delta_{\tau }/2\right)}^{\tau +\left(\Delta_{\tau }/2\right)}x\left(t_{s}\right)y\left(t_{s}+t_{i}\right)$$

where *N*_*x*_ and *N*_*y*_ are the number of spikes in trains *x* and *y*, respectively; *t*_*s*_ is the spike occurrence time in train *x*; and Δ_τ_ is the time window in which synchronous spikes occur in train *y*.

Synaptic connections were detected, and the directions were defined by the polarity of the detected peaks (negative peaks represent presynaptic connections, and positive peaks represent postsynaptic connections) (English et al., 2017). The average cross-correlation of learning neurons in the network plus 3 times the average standard deviation was selected as the threshold to construct the network. The network indicators were calculated as follows:

Synchrony: For each spike, the synchrony was calculated as the duration between that spike and the closest spike in the reference event. This analysis was useful for identifying times when several neurons were in sync with the reference neuron. First, we selected the inverted distance; high values indicated spikes that were close to each other.

In the following equations, *N* is the set of all in the network, and n is the number of nodes; *L* is the set of all links in the network, and *l* is the number of links; (*i, j*) is a link between nodes *i* and *j*, (*i, j∈N*); and *a*_*ij*_ is the connection status between *i* and *j*.

Degree:


(3) $$k_{i}=\sum_{j\in N}a_{ij}$$

Network density:


(4) $$d=\frac{2L}{N\left(N-1\right)}$$

Global efficiency:


(5) $$E=\frac{1}{n}\sum_{i\in N}E_{i}=\frac{1}{n}\sum_{i\in N}\frac{\sum_{j\in N,j\neq i}d_{ij}^{-1}}{n-1}$$

Modularity:


(6) $$Q=\sum_{u\in M}\left[e_{uu}-{\left(\sum_{v\in M}e_{uv}\right)}^{2}\right]$$

where the network was fully subdivided into a set of non-overlapping modules *M*, and *e*_*uv*_ is the proportion of all links that connect nodes in module *u* with nodes in module *v*.

The betweenness centrality of node *i* was calculated as follows:


(7) $$b_{i}=\frac{1}{\left(n-1\right)\left(n-2\right)}\sum_{\begin{array}{c} h,j\in N\\ h\neq j,h\neq i.j\neq i \end{array}}\frac{\rho_{hj}^{\left(i\right)}}{\rho_{hj}}$$

where ρ_*hj*_ is the number of shortest paths between *h* and *j*, and ρ_*hj*_*(i)* is the number of shortest paths between *h* and *j* that pass through *i*.

Small-world:


(8) $$S=\frac{C/C_{rand}}{L/L_{rand}}$$

where *C* and *C*_*rand*_ are the clustering coefficients, and *L* and *L*_*rand*_ are the characteristic path lengths of the respective tested network and a random network.

## Data availability statement

The raw data supporting the conclusions of this article will be made available by the authors, without undue reservation.

## Author contributions

XJ performed research and analyzed the data. XJ, XL, and WS designed research and wrote the manuscript. NH, JS, XF, CC, YW, LC, and HQ helped complete the experiment. All authors contributed to the article and approved the submitted version.

## Supplementary material

Supplementary Figure 1 The influence of learning on the spontaneous activity of the cultured neuron network. **(A)** The number of bursts within 1 min in the neuronal network was assessed in the learning phase. **(B)** The number of participating electrodes in each burst in the learning phase. **(C)** The duration of each burst in the learning phase (*n* = 5 cultures. **p* < 0.05 vs. Cycle 1,^**^*p* < 0.01 vs. Cycle 1).

Supplementary Figure 2 The effect of bicuculline on stimulus-evoked response in learning populations in cultured neuron networks. **(A)** The R/S ratio of learning populations and all neurons within 200 ms of the stimulus in cultured neuronal networks from different groups. **(B)** The RT of learning populations and global populations within 200 ms of the stimulus in cultured neuronal networks in the different groups. **(C)** The amplitude of evoked responses in learning populations and global populations within 200 ms of the stimulus in cultured neuronal networks from different groups. **(D)** Effects of bicuculline on the global efficiency of stimulus-evoked responses. **(E)** Effects of bicuculline on the network density of stimulus-evoked responses. **(F)** Effects of bicuculline on small-world stimulus-evoked responses. The average inverse shortest path length is a measure known as the global efficiency and describes the parallel information processing ability of the network. Network density can be used to characterize the density of interconnected edges between nodes in a network. Small-world networks combine the presence of functionally specialized (segregated) modules with a robust number of intermodular (integrating) links (*n* = 5 cultures. **p* < 0.05 vs. learning population control, ^**^*p* < 0.01 vs. learning population control. #*p* < 0.05 vs. learning population bicuculline, ##*p* < 0.01 vs. learning population bicuculline. *p* < 0.05 vs. learning population wash, △*p* < 0.01 vs. learning population wash).

Supplementary Figure 3 The effect of bicuculline on the spontaneous activity of learning populations in cultured neuron networks. **(A)** The correlation of spontaneous activity between learning populations and global populations in cultured neuronal networks from different groups. **(B)** The synchrony of spontaneous activity between learning populations and global populations in cultured neuronal networks from different groups. **(C)** Effects of bicuculline on the network density of spontaneous activities. **(D)** Effects of bicuculline on small-world spontaneous activity. **(E)** Effects of bicuculline on the global efficiency of spontaneous activities. **(F)** Effects of bicuculline on the number of spontaneous activity bursts (time: 60 s). **(G)** Numbers of electrodes per burst in cultured neuronal networks from different groups. **(H)** Mean burst duration in cultured neuronal networks from different groups (*n* = 5 cultures. **p* < 0.05 vs. learning neuron control, ^**^*p* < 0.01 vs. learning neuron control. ^#^*p* < 0.05 vs. learning neuron bicuculline, ^##^*p* < 0.01 vs. learning neuron bicuculline).
