# Supplementary figures and images for: Learning populations with hubs govern the initiation and propagation of spontaneous bursts in neuronal networks after learning
Source: Front Neurosci. 2022 Aug 18;16:854199. doi: 10.3389/fnins.2022.854199 (PMC9433803; doi:10.3389/fnins.2022.854199)

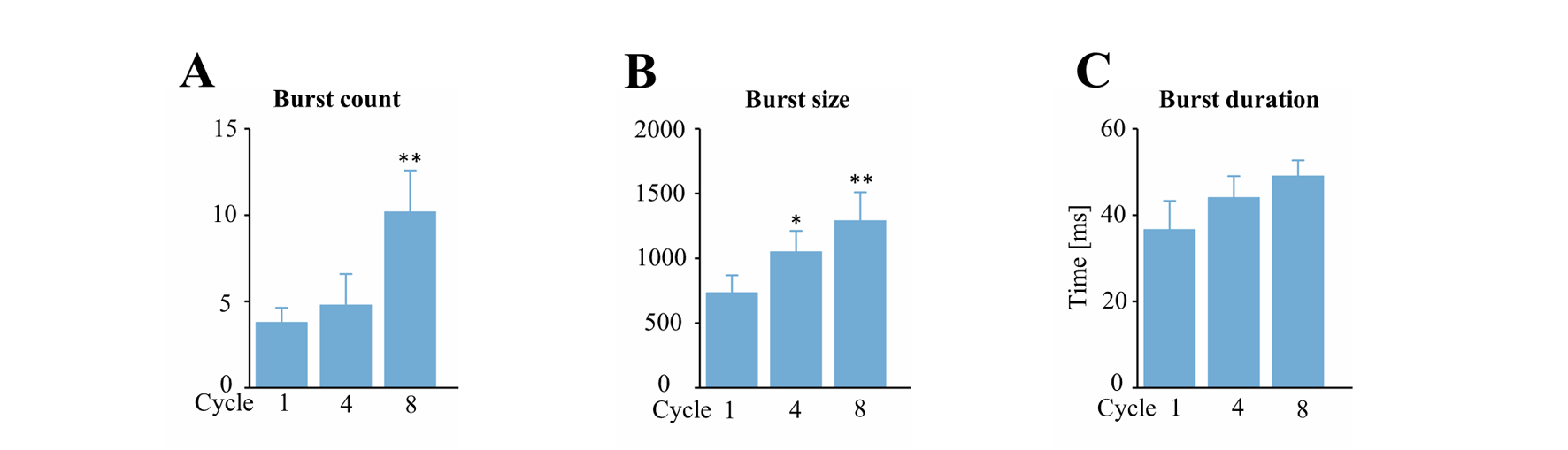

Supplement: Supplementary Figure 1 — The influence of learning on the spontaneous activity of the cultured neuron network. (A) The number of bursts within 1 min in the neuronal network was assessed in the learning phase. (B) The number of participating electrodes in each burst in the learning phase. (C) The duration of each burst in the learning phase (n = 5 cultures. *p < 0.05 vs. Cycle 1,**p < 0.01 vs. Cycle 1). [file Image_1.TIF]

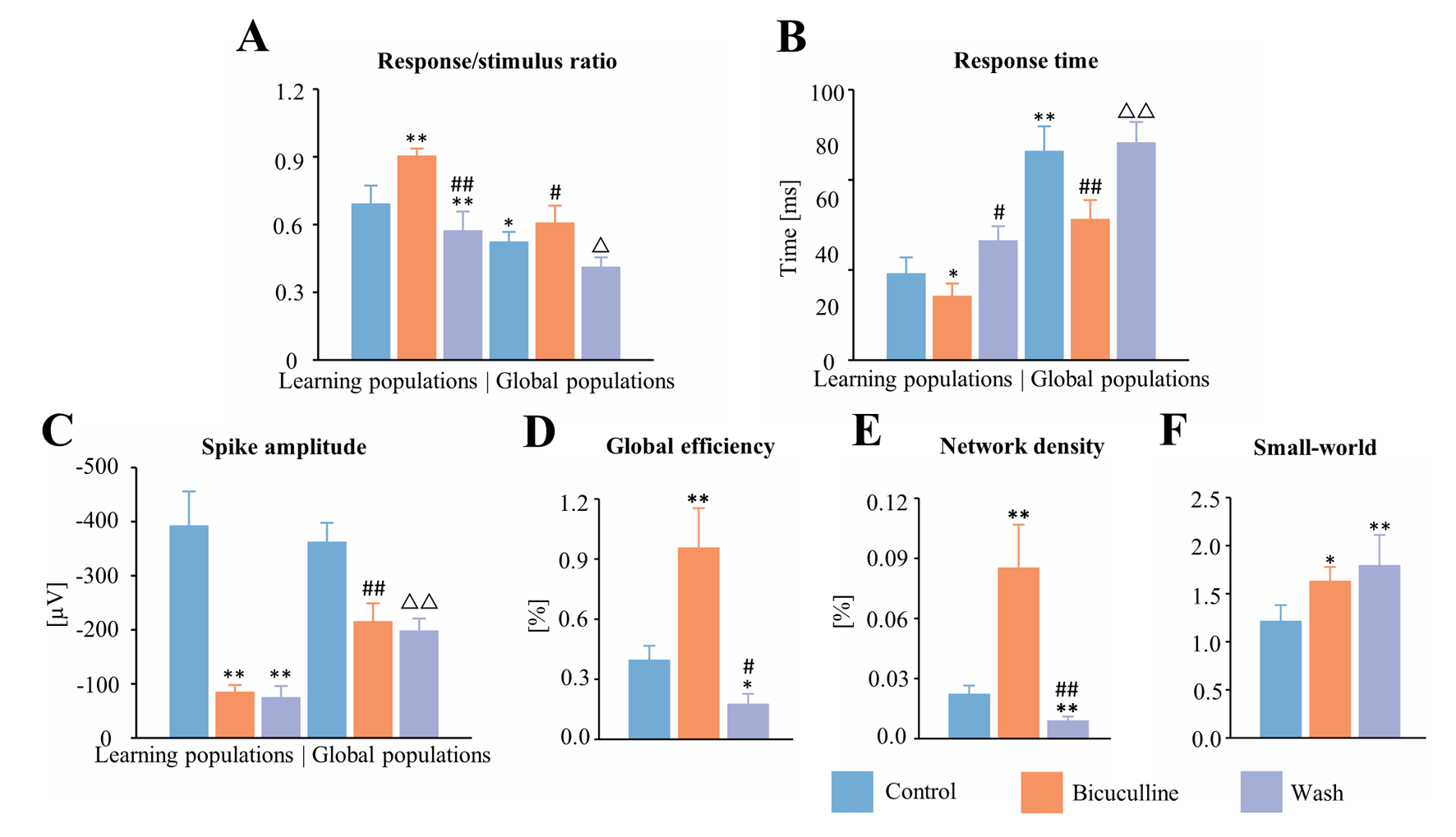

Supplement: Supplementary Figure 2 — The effect of bicuculline on stimulus-evoked response in learning populations in cultured neuron networks. (A) The R/S ratio of learning populations and all neurons within 200 ms of the stimulus in cultured neuronal networks from different groups. (B) The RT of learning populations and global populations within 200 ms of the stimulus in cultured neuronal networks in the different groups. (C) The amplitude of evoked responses in learning populations and global populations within 200 ms of the stimulus in cultured neuronal networks from different groups. (D) Effects of bicuculline on the global efficiency of stimulus-evoked responses. (E) Effects of bicuculline on the network density of stimulus-evoked responses. (F) Effects of bicuculline on small-world stimulus-evoked responses. The average inverse shortest path length is a measure known as the global efficiency and describes the parallel information processing ability of the network. Network density can be used to characterize the density of interconnected edges between nodes in a network. Small-world networks combine the presence of functionally specialized (segregated) modules with a robust number of intermodular (integrating) links (n = 5 cultures. *p < 0.05 vs. learning population control, **p < 0.01 vs. learning population control. #p < 0.05 vs. learning population bicuculline, ##p < 0.01 vs. learning population bicuculline. p < 0.05 vs. learning population wash, △p < 0.01 vs. learning population wash). [file Image_2.TIF]

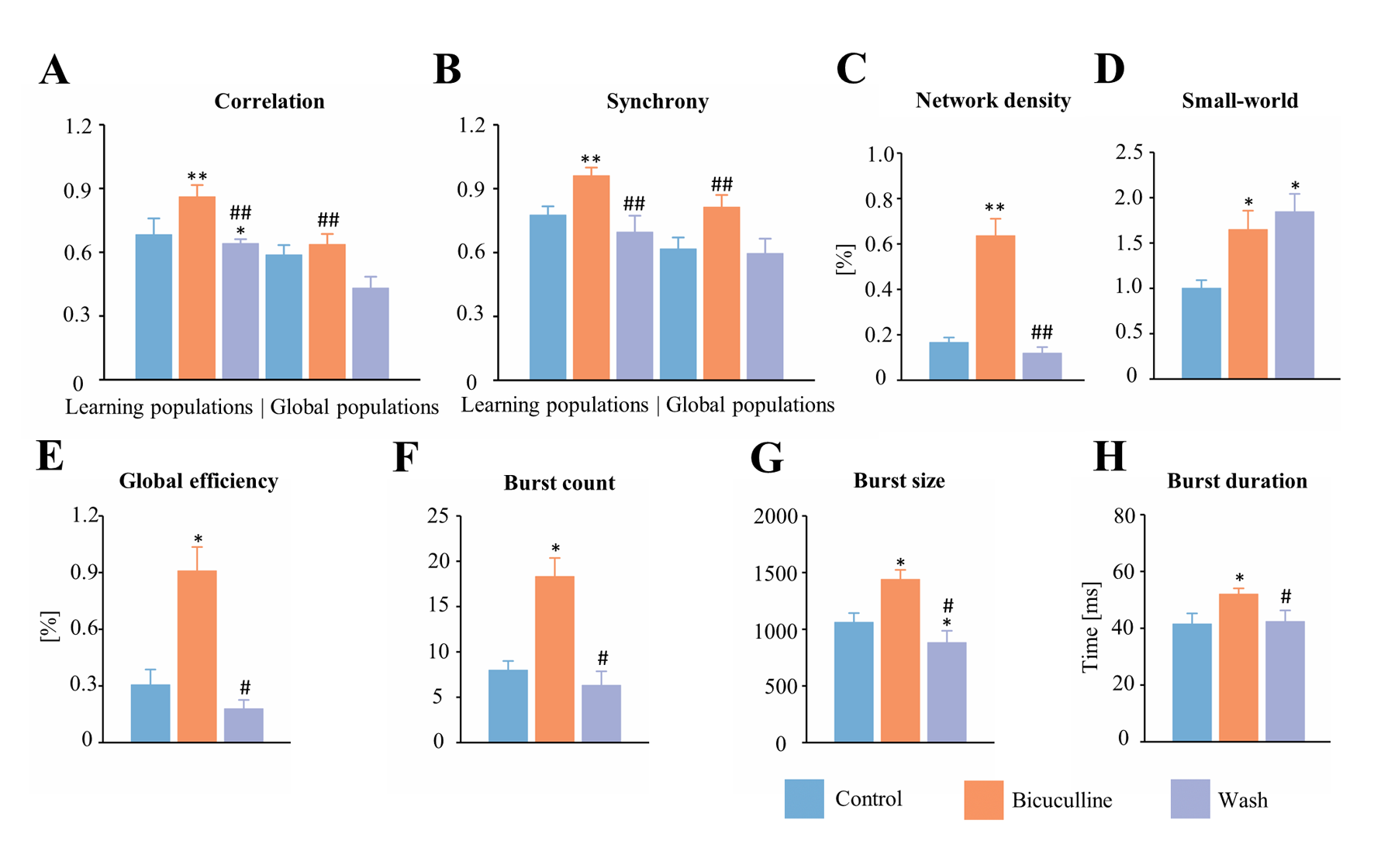

Supplement: Supplementary Figure 3 — The effect of bicuculline on the spontaneous activity of learning populations in cultured neuron networks. (A) The correlation of spontaneous activity between learning populations and global populations in cultured neuronal networks from different groups. (B) The synchrony of spontaneous activity between learning populations and global populations in cultured neuronal networks from different groups. (C) Effects of bicuculline on the network density of spontaneous activities. (D) Effects of bicuculline on small-world spontaneous activity. (E) Effects of bicuculline on the global efficiency of spontaneous activities. (F) Effects of bicuculline on the number of spontaneous activity bursts (time: 60 s). (G) Numbers of electrodes per burst in cultured neuronal networks from different groups. (H) Mean burst duration in cultured neuronal networks from different groups (n = 5 cultures. *p < 0.05 vs. learning neuron control, **p < 0.01 vs. learning neuron control. #p < 0.05 vs. learning neuron bicuculline, ##p < 0.01 vs. learning neuron bicuculline). [file Image_3.TIF]
